#  A Digital Pornography Literacy Resource Co-Designed With Vulnerable Young People: Development of "The Gist"

**DOI:** 10.2196/15964

**Published:** 2020-06-01

**Authors:** Angela C Davis, Cassandra JC Wright, Stacey Murphy, Paul Dietze, Meredith J Temple-Smith, Margaret E Hellard, Megan SC Lim

**Affiliations:** 1 Burnet Institute Melbourne Australia; 2 Department of Epidemiology and Preventive Medicine Monash University Melbourne Australia; 3 Centre for Alcohol Policy Research La Trobe University Melbourne Australia; 4 Sheda Melbourne Australia; 5 Department of General Practice Melbourne Medical School University of Melbourne Melbourne Australia; 6 Doherty Institute and School of Population and Global Health University of Melbourne Melbourne Australia; 7 Department of Infectious Diseases The Alfred Hospital Melbourne Australia; 8 Melbourne School of Population and Global Health Department of General Practice University of Melbourne Melbourne Australia

**Keywords:** co-design, pornography literacy, sexual health, sex education

## Abstract

**Background:**

The impact of viewing pornography at a young age on the sexual health of subgroups of young people is an important public health issue. However, the topic is complex and extremely sensitive, and best practices for research and harm reduction are yet to be defined. Drawing on cross-disciplinary approaches, such as co-design, is one way to achieve a better understanding of the issue among vulnerable young people and to create needs-based and evidence-informed digital resources to promote pornography literacy.

**Objective:**

The objective of this study was to co-design a relevant, usable, and acceptable digital prototype to address the pornography literacy needs of vulnerable young people.

**Methods:**

In total, 17 young people aged between 14 and 23 years who were engaged in youth services programs or alternative education programs were recruited to participate in 4 co-design workshops with a multidisciplinary design team.

**Results:**

Although the participants could identify problems with pornography and critique its messages, they lacked the information to understand alternative healthy attitudes and behaviors. A digital resource that provides detailed and practical information about sex, sexual ethics, and relationships may help vulnerable young people to identify and contrast with any problematic messages they receive from both pornography and society. Embedding this information with pornography literacy messages may be a more effective way of addressing underlying attitudes. Acknowledging information-seeking patterns and leveraging user interaction patterns from commonly used digital platforms among users may enhance engagement with resources. Importantly, digital platforms are perceived among this group as a source of anonymous secondary information but would not be organically accessed among this group without face-to-face conversations as an access point.

**Conclusions:**

This paper highlights the potential for pornography literacy to be embedded within real and practical information about having sex, navigating sexuality, and healthy relationships. The study findings include important recommendations for the conceptualization of digital pornography literacy programs and opportunities for cross-disciplinary co-design research to address complex and emerging health issues.

## Introduction

### Background

Young people’s increasing access to free web-based pornography has led to concerns over its impact on their attitudes and behavior [1]. Pornography is defined as sexually explicit pictures, texts, or other material created to cause sexual arousal [2]. However, in reality, web-based pornography includes a wide variety of material and genres that depict diverse sexual scenes [3-5]. The literature suggests that condom use in heterosexual pornography is rare (2%-3%) and that gender inequality in mainstream online videos is common [3,5,6]. For instance, female actors were found to be significantly more likely to be shown being dominated or degraded [3] and were four times less likely than male actors to be shown achieving orgasm [4]. Recent research with 15- to 29-year-old Australians found that 90% had ever viewed pornography, with 81% of young men and 22% of young women reporting at least weekly use [7]. Pornography exposure is associated with sexual risk factors, including decreased age of first sexual experience, more lifetime sexual partners, and not using a condom during the last sexual encounter [8-10]. Exposure has been associated with increased sexual aggression and sexist attitudes [11], and cross-sectional studies report links with relationship problems and depression [12-16]. However, harmful effects have not consistently been reported in research, with some studies showing mixed findings [17]. Given the scale of the issue, it is a public health imperative to better understand how viewing this content frequently and from a young age may impact on sexual scripts, which include the knowledge, beliefs, and attitudes that guide sexual decision making [18,19]. However, the topic is evolving, complex, and extremely sensitive, and best practices for research and harm reduction are yet to be defined. Drawing on cross-disciplinary approaches, such as co-design, is one way to achieve a better understanding of the issue among young people and to create needs-based and evidence-informed responses.

### Vulnerable Young People at Risk

Pornography is not homogenous, nor are its audiences [20]. Informed by the social cognitive theory, researchers have suggested that there may be differential effects of viewing pornography, influenced by factors such as the type of pornography viewed; the viewer’s age, gender, and sexuality; preheld beliefs; and access to alternative sources of information [10,16,21,22]. How these factors influence the impact of pornography on knowledge, attitudes, and behaviors is beginning to be explored among subgroups of young people to understand risk factors for harm. A 2018 cross-sectional survey of 15- to 29-year-old heterosexual Australians (n=517) by our group found that young women (n=320) were significantly more likely than young men (n=197) to report having seen violent or degrading behaviors toward women [23]. We hypothesized that gendered experiences may influence how young audiences interpret pornographic images rather than indicate pornography preferences [23]. A 2015 study of economically disadvantaged, urban-residing black and Hispanic youth (n=72) identified an association between regular pornography use and dating violence [10]. Other studies have found that pornography can play an important role in the sexual development of lesbian, gay, bisexual, transsexual, queer, and intersex plus (LGBTQI+) young people in the context of limited access to alternative representations and overrepresentation of heteronormative sex [24,25]. However, few studies have explored how this may negatively influence ideas about sex, gender, and sexuality for young LGBTQI+ people exposed to fetishized representations of sexuality [26]. In summary, there may be young people who are at greater risk of harms from pornography as a result of the sociocultural and environmental contexts in which they view it. For instance, young people who do not consistently attend school, experience family conflict, identify as gender or sexuality diverse, or are from culturally and linguistically diverse backgrounds may have specific experiences and less access to relevant sexuality and relationships education, including pornography literacy. Understanding how these subgroups of young people experience pornography and its effects is vital to creating effective health promotion responses.

### Pornography Literacy

Despite the evidence of the impacts of pornography on young people being underdeveloped, interventions to reduce its potential harms are being funded and produced [27-30]. Pornography literacy has been advocated as a harm reduction approach. On the basis of the media literacy theory [31], the key aims of pornography literacy approaches are to teach young people skills to critically analyze the messages in pornography, to increase their understanding of risks of exposure, and to encourage them to hold critical attitudes toward viewing the content and its messages [21,29,31,32]. However, there is little consensus about what pornography literacy education programs should include or how they should be delivered [33]. One of the few studies that explored the pornography literacy needs of young people used participatory research methods to identify information needs among Irish young adults aged 18 to 29 years [33]. Dawson identified pornography literacy priorities, including communication and consent, body image, reality of real-life sex, pleasure, information about the fetishization of gender and sexually diverse groups, and reducing shame over pornography use [33].

Importantly, current literature is inconclusive about the effectiveness of pornography literacy to reduce pornography-related harms among young people [32,34,35]. A small body of the literature suggests that participation in media literacy may help young people to develop skills to critique media messages and develop realistic expectations [33]. For instance, a representative panel survey of Dutch adolescents found an association between receiving school-based pornography literacy education and reduced strength in the relationship between pornography and stereotypical sexual attitudes [35]. However, the study did not define pornography literacy, relying on self-reporting of receiving any school-based education about pornography [35]. A recent preliminary study of 24 college students (aged 15-24 years) assessed the feasibility and effectiveness of a class-based pornography literacy program; it found that pornography-related knowledge increased posttest and some attitudinal shifts resulted [32]. These programs have been implemented and evaluated among young people engaged through formal in-person education and, thus, may not be applicable to those not in formal education settings. To date, there are no publicly available online or digital pornography literacy resources that specifically target vulnerable young people. Research has suggested that online resources could complement and enhance information engagement [33] and are most valuable to young people who lack alternative information, including those with diverse gender and sexual identities [36]. As such, understanding the experience, needs, and wants of subgroups that may be particularly vulnerable to the effects of viewing pornography will assist in developing responsive and equitable digital resources.

### Human-Centered Design

Human-centered design and design thinking approaches involve prioritizing users throughout the creation of a product or service rather than at the start or end [37,38]. As a tool of human-centered design and design thinking, co-design methods involve bringing together key users, designers, and subject matter experts who participate in iterative workshops to understand and empathize with users. This enables them to define key issues and opportunities, to develop ideas to overcome issues, and to design solutions. Using creative and generative activities enables engagement with lived experiences and perspectives in ways that can overcome limitations of verbal communication where topics are complex or sensitive [39,40]. If implemented well, co-design methods can provide participants with time and space to actually think about the issue as they create, form, and question opinions and reflect on theirs and others’ experiences in a safe and productive setting. Creating, role playing, and interaction between facilitators and participants can shift power dynamics and create trust and mutuality to understand and define sensitive issues. Importantly, involving users who are experts of their own experience as co-designers of potential solutions can help overcome the limitations of relying on traditional formative research methods to create health interventions [41].

Despite the seemingly obvious alignment of design and health disciplines, recognized challenges for cross-disciplinary collaboration exist [40,42]. For instance, it has been acknowledged that health research principles such as control, generalizability, reduction of bias, and replicability are in contrast with co-design processes that are necessarily creative, iterative, and flexible to account for the needs of users [40]. Furthermore, challenges of bringing together cross-disciplinary expertise central to co-design processes, such as time, resources, and willingness to compromise on disciplinary approaches could influence those considering this approach. Even the process of communicating co-design processes and findings falls outside of qualitative research conventions. Despite these challenges, the potential for the co-design method to radically contribute to the field of public health and health promotion is immense. For instance, there has been growing recognition of the potential for this method to increase the effectiveness of health promotion practices, particularly where issues are complex, sensitive, and ill-defined [37,39,42,43]. This being the case, these methods are particularly relevant for addressing the impact of web-based pornography on vulnerable young people’s sexual health and their preferences for pornography literacy education.

### This Study

This study aimed to co-design a relevant, usable, and acceptable digital prototype to improve pornography literacy among vulnerable young people. The project involved a cross-disciplinary co-design, including vulnerable young people, service providers, public health researchers, design researchers, user interface designers, and web developers.

## Methods

### Participants

A total of 17 young people aged between 14 and 23 years participated in the multiphase co-design workshops. The participants included 5 young women, 1 gender nonbinary person, and 11 young men.

### Procedure

Young people were eligible if they were currently engaged with one of our partner youth service providers, were aged 14 to 24 years, and had experiences or identified with of at least one of the following: fragmented school attendance or disengagement from mainstream education (irregular attendance or participation in alternative curriculums such as vocational or applied learning); limited access to relevant education on sex, sexuality, and relationships (ie, for young people who identify as LGBTQI+); experience of family conflict or breakdown; and culturally and linguistically diverse (CALD) backgrounds.

We partnered with youth services, education providers, and primary health care providers to embed us in their existing programs for young people. This included youth groups and alternative education programs for young people disengaged from mainstream schools. A written information and consent form was developed and was accompanied by a short video to facilitate information and consent for people with low literacy skills. Project information was housed on a website to ensure that community members and potential co-designers could access further information about the project or contact our team. Informed consent was obtained from participants aged 16 years and older. For participants aged 15 years, we followed consenting practices for mature minors [44] of partner organizations, which involved independently consenting those deemed mature minors and seeking parental consent for others. Parental consent was obtained for participants younger than 15 years. The participants received an Aus $40 (US $29) gift voucher for every 3-hour workshop, with a bonus Aus $20 (US $12.11) voucher for those who attended all workshops. Facilitators, participants, and youth workers communicated about any sensitive or traumatic issues that may have emerged during activities. Participants were given the opportunity to change activities, observe, or leave it any time and to debrief or access relevant services after each workshop. Ethics approval for the research was granted by the Alfred Health Human Research Ethics Committee (373/18).

### Participant Characteristics

A total of 17 young people participated in the co-design workshops, including 5 females, 1 gender nonbinary person, and 11 males. Participants ranged in age from 14 to 23 years (mean 16.5 years). Furthermore, 6 participants were from CALD backgrounds (English as a second language), 5 identified as LGBTQI+, and 2 were living with an intellectual disability. Over one-third of our sample was disengaged from mainstream schooling and had experiences of fragmented education. A similar proportion had experiences of family conflict or breakdown.

### Co-Design Workshops

We conducted a total of 12 co-design workshops across 3 groups. Workshops were designed across 4 phases adapted from design thinking [37]: *understand, define, ideate,* and *design*. Workshops were conducted in known and safe spaces, including schools, community centers, and youth services. Two design researchers (1 male and 1 female) and a public health researcher (female) facilitated all the workshops. Youth workers and teachers were present on site but not in the room during the workshops. Each workshop ran for 3 hours. Workshops were audio-recorded, and note-takers recorded key conversations, observations, and interactions.

The first workshop focused on introducing the process, building trust and rapport, creating shared boundaries and safety, and beginning the process of getting to know our users and their information preferences. Activities were designed to create opportunities even during the introduction and icebreakers to understand more about our user’s lives, aspirations, motivations, and challenges to help us walk in their shoes. The second workshop focused on creating opportunities for our participants to reflect on their thoughts, feelings, and experiences of sex, relationships, and pornography and to enable them to define key issues or problems, hence identifying information needs. We were conscious of creating space for participants to speak openly, using their own language about issues they may feel unsafe or embarrassed to talk about in the workshop setting. We designed activities that could be fully engaged with either anonymously or in the third person. The aim of the third workshop was to review young participants’ definition of the problem and position our participants as *solution designers*. This involved building participants’ understanding of design processes and tools and belief in themselves as *designers*, while simultaneously exploring their preferences for solutions. Activities facilitated the ideation of *blue-sky* ideas to help address issues defined throughout the previous workshops. The aim of the fourth workshop was to enable participants to develop their ideas into digital design solutions and to understand their brand values and aesthetic preferences for the user interface design. Participants used digital wireframes to map out their solution, features, and interactivity.

### Workshop Activities

**Figure 1:**
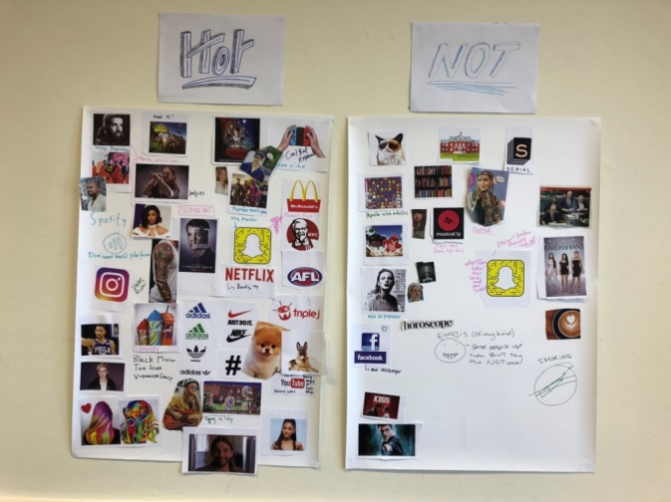
Hot or Not? Collage.

During activity design, we considered our participants’ interests, abilities, and challenges and, our design practices. Activities prioritized creativity, shared understanding, empathy, and safety. In the first workshop, participants created personas of other young people that could be used for third-person discussions to avoid over disclosure. Across the workshops, each activity was built on the topics and outcomes from previous activities. This allowed participants to work into this complex issue and to increase trust and engagement as the activities progressed. Activities were generative to create spaces for participants to embrace creativity and to reflect on the issues [45]. Informed by previous co-design projects engaging with young people [39,43], we used a range of creative tools, including Lego, collage (Figure 1), community-mapping, drawing, music, and movie making. For instance, one activity involved participants creating a storyboard based on a scenario related to sex, relationships, or porn. They used personas to explore what the people in the scenario thought, felt, and did. Participants used Lego to create each scene of their movie along with speech bubbles. Each small group used a preloaded *stop motion* video app [46] to create a short movie, which they recorded and played back to the group for discussion. Activities positioned participants as designers throughout the process from understanding and defining the issue right through to the design of actual solutions. We conducted solution prototyping using pop culture references, such as superheroes, to leverage universal recognition and engagement with the Marvel comic franchise. A full description of our activity development and implementation is described in a research protocol and can be accessed on request.

### Data Analysis

Data generated from activities included (1) the products of these activities that were considered *assets* that we could analyze; (2) our debrief notes that recorded conversations, interactions, and observations; and (3) verbal recordings of participants’ discussions during the activities. All findings are developed based on insights drawn from across activities.

#### Co-Design Synthesis

An iterative human-centered design data synthesis using *affinity mapping* guided the analysis of data [47]. Step 1 involved extracting data from the assets the participants created, workshop recordings, transcription notes, and debrief notes. Step 2 involved sorting the data into several deductive themes that were clustered and coded together to establish subthemes. This step involved building consensus among the team through disagreement and debate. Step 3 involved *collaborative sense-making* where the research team interpreted the themes and subthemes into design insights [47]. For instance, the theme of youth trauma was evident across the workshop; in our data synthesis phase, we translated this into a design recommendation of needing our resource to be trauma-informed, which we placed under the *information preferences* branch of design considerations. We identified how our insights were connected to create a picture of our users’ needs and challenges for the design. Each stage of the synthesis process included researchers AD and SM, and the final steps also included researcher CW and members of the design team.

#### Prototype Design

Human-centered design practices guided our process for bringing together the needs and wants of our users with best practice in accessible design and health promotion. The multidisciplinary team of designers and researchers were involved in the final stages of the synthesis and then participated in a process called Design Sprints. The process included ideating solutions based on user personas (available on request), on participants’ design solutions created during co-design workshops, and on the response to the information needs and preferences of each user persona. Multiple rounds of ideation and design took place before a set of design recommendations for the prototype was developed. On the basis of the feedback from participants, key stakeholders, and external subject matter experts, these recommendations were developed into a functioning digital prototype of the solution for testing and evaluation (to take place in an independent study).

## Results

### Design Considerations

A summary of key design considerations (*information needs* and *information preferences)* identified through data synthesis and translation of co-design insights have been outlined as follows, after which the full description of the final prototype design has been provided.

### Information Needs

#### Pornography Literacy

**Figure 2:**
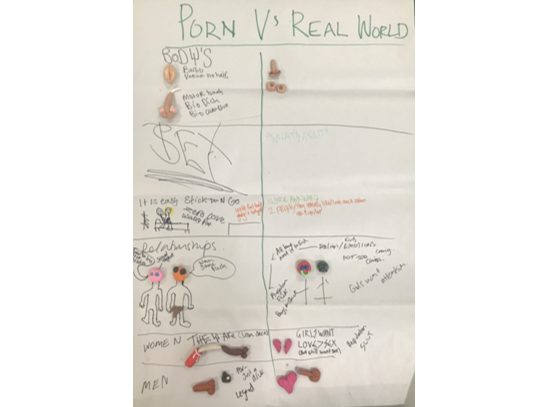
Porn world vs. real world activity creation.

Participants defined pornography as *anything you can see on a porn site*, including porn videos, images, explicit pop-up advertisements (eg, for penis enlargements), and third-party services (eg, for paid sex). Activities and discussion among our users illustrated that they understood pornography as obviously unrealistic and could already critique its messages. Young women identified concerns over the representation of women’s bodies, gender stereotypes such as *women who like sex are sluts,* and the reinforcement of rape myth acceptance. In contrast, young men and young transmen commonly identified problematic messages about bodies, including male bodies, male sexual performance, and normalization of the objectification of women, such as their representation as *i* (Figure 2). Despite being able to identify problematic messages, young men did not link them to their own attitudes or behaviors. Rather, some explicitly stated that knowing pornography was “fake” made them “porn proof.” Others explained “we don’t all watch Harry Potter and want to become wizards” to articulate to the group how their understanding of pornography as *fantasy* meant that they could watch it without having their attitudes or behaviors affected.

**Figure 3:**
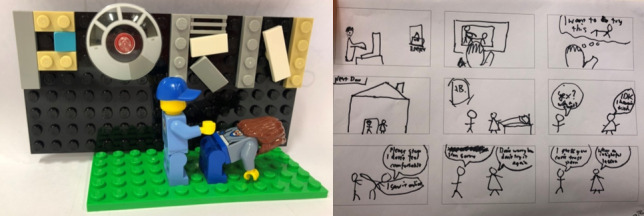
Stop motion movie scene and storyboard activity creation.

In contrast to these claims, young men created scenarios in which watching pornography did impact their behavior. For instance, a pair of participants created a stop-motion Lego movie illustrating how a character watched pornography and, after viewing anal sex, decides to try it with his girlfriend who is uncertain about it (Figure 3). In the scenario, the girl asks the guy to stop because she does not feel comfortable. He complies, only saying “I saw it online,” and no harm is perceived to be done. The scenario and subsequent discussions revealed a contradiction to our participants’ statements that watching porn did not affect their attitudes or behavior in any way and potential barriers to addressing impacts by focusing on the potential harms from porn.

#### Beyond a Problem With Porn

Activities revealed that our users lacked information and skills to be able to contrast problematic messages from pornography with alternative ways of thinking or behaving. For instance, although participants could identify that representations of pleasure and consent in pornography were limited, there was little consensus among the groups about how to make sex pleasurable for all people and what constitutes consent or ethical sexual practices. This was particularly problematic for LGBTQI+ young people who experienced even less access to relevant representations of sex and sexuality outside of porn. Specific information gaps identified across groups included pleasurable sex positions, sexual pleasure for young women and trans young people, sexual pressure, consent, safe sex, and healthy relationships. Without alternative information and frameworks to guide their decision making and preferences, our participants were concerned with their ability to navigate both the “basics” and the “grey” areas of sex and relationships.

Furthermore, our users identified how messages from pornography can be compounded by messages they are exposed to in other domains. For young women in particular, problematic messages that they are exposed to in pornography are not always different from those in popular culture and society. For instance, some talked about the messages they are exposed to on Instagram about the ideal female body and sexuality. Some young men also articulated concerns about unhealthy behaviors they had seen modeled by family members such as a “drunk uncle” who makes people feel uncomfortable with his behavior. These insights highlight potential problems with creating a resource centered on pornography as an issue without addressing the information gaps and underlying social norms that permeate young people’s lives.

### Information Preferences

#### Reactive Information Seeking

In general, our users were unlikely to proactively seek out information, especially about sex, relationships, and porn, unless prompted by experience or contradiction to preheld knowledge. Rather, they wait until they have a negative or confusing experience or until their beliefs are contradicted to *Google it* This was illustrated during workshops when participants would often talk to each other about the issues raised through activities. On one occasion, 2 participants debated if girls can orgasm during penetrative sex. After discussion and disagreement, they pulled out their phones and searched for an answer. Both confirmed their own assumptions by finding something online to illustrate their point, highlighting that once they seek out information, many look to confirm or validate their existing opinions or beliefs. Importantly, neither participant found quality or evidence-based information; we found that few participants had the knowledge or skills to research high-quality information in practice. This has important implications for designing a resource on this topic.

#### Trauma-Informed

Participant’s experiences of trauma affected their engagement with these sensitive topics. Some who had experienced sexually related trauma found overtly sex-positive sex education content retraumatizing. Other participants specified finding visual and descriptive content about sexually transmitted infections and detailed real-life stories of sexual violence to be traumatizing. As stated by a young man who said:

You don’t want to see like diseased bodies (sic). It’s just gross. But at the same time, you want to know about what happens. It’s the same with like stories about sexual assault. I’m sorry but they are just hard to read sometimes.

Although there was an understanding of the need to be aware of behaviors and consequences, young people’s personal and online experiences of this educational content sometimes resulted in them withdrawing from engagement altogether. The sentiments demonstrated the fine balance between being direct and positive about sex without traumatizing or downplaying the sometimes negative experiences of sex among our users.

#### Prototype

**Figure 4:**
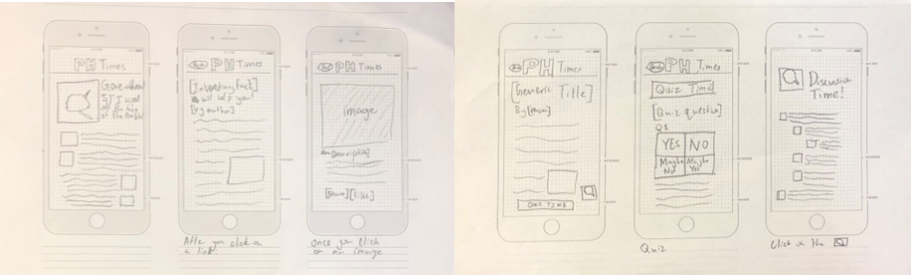
A digital wireframe of a solution concept created by a young co-designer during prototyping.

On the basis of the results described earlier, a high-fidelity digital prototype was designed by a cross-disciplinary team of researchers and designers. A digital prototype is a way of creating a working example of a product that is tangible enough that it can be tested with a larger population and further developed based on new learnings. The design processes leveraged the solution designs (Figure 4), needs, and wants of co-design participants that were aligned with health promotion and design accessibility best practice [48]. The digital prototype developed, called *The Gist,* is described in the following sections with reference to the rationale for content (relevance and acceptability) and design (usability and accessibility).

#### The Gist

**Figure 5:**
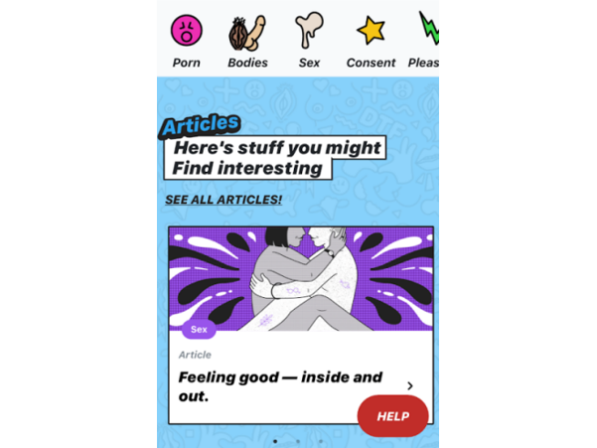
The Gist web-based mobile application prototype home screen.

The Gist (Figure 5) is an interactive mobile-first web-based app prototype. The aim of the design is to provide vulnerable young people with an interesting and easy way to take control of their sexual health and well-being in the context of normalized pornography use.

#### Content

**Figure 6:**
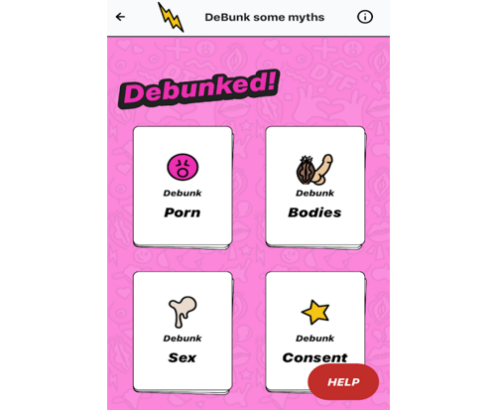
The Gist ‘Debunked’ activity to challenge pre-held knowledge and attitudes.

The Gist content provides alternative information that our users want and are not getting from porn, society, or mainstream education; it aims to develop healthy ideas about relationships, sex, and bodies. Drawing on concepts underpinning ethical sex, as described by Carmody and Ovendon [49], the resource aims to provide practical steps that users can follow to develop skills and preferences and help them to consider how their choices impact on themselves and others. For instance, practical information about sex is provided to help them develop an understanding of their own needs and their partners’ needs. This content is linked with steps they can take to identify, understand, and communicate these needs. Critical pornography literacy is built into messages by contrasting this alternative information with problematic messages present in pornography and society. This shifts the focus away from the *problems with porn* toward meeting our users’ identified needs, while still enhancing the critical literacy of problematic representations of sex, sexuality, and gender. This includes information about sex positions, finding out what you like, sexual pleasure, sexual arousal, bodies, relationships, consent, and porn. The content balances direct, challenging, and informative articles with opportunities to build and test skills through interactive elements such as quizzes (Figure 6). Content is presented in gender- and sexuality-neutral ways in response to enable young people to explore information in an inclusive environment.

#### User Interface and Experience Design

**Figure 7:**
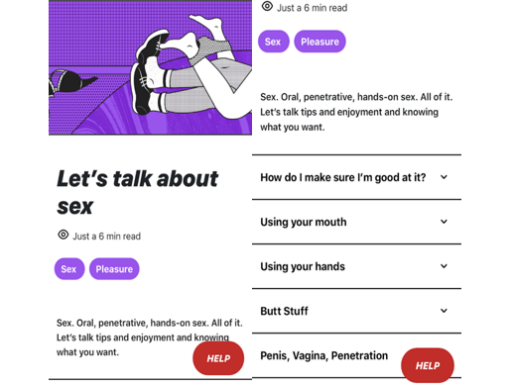
The Gist article structure utilizing progressive disclosure to increase user control.

The Gist brand is designed with a bold typography in keeping with our audiences’ desire for alternative and nonmainstream products, and a *retro* 90s design feel. The name, *The Gist*, refers to the process of getting to know the idea or main point of a concept. It was selected for the prototype through testing with co-design participants. The user interface leverages our users’ reactive information-seeking habits, and quick content for browsing aims to surprise or contradict our users’ ideas and experiences motivating them to seek out more information. Short *gists* or facts are linked through to deeper exploration of the issues to enable in-app learning to progress once a user is engaged. Multiformat content also leverages user experience (UX) and interaction patterns from apps our users frequently engage with such as Spotify, Instagram Stories, and YouTube. This enhances the familiarity and usability of The Gist for our target population. Capitalizing on our users current UX patterns, the app enables continuous scrolling for breadth of engagement and continuous links to further content for depth of engagement [48]. Detailed articles utilize progressive disclosure (informative headlines with expandable information sections; for example, Figure 7). Users are provided with staggered content that can be engaged with if elected. This allows young people to take control of what they see and to elect not be exposed to information that they are uncomfortable with. This is based on insights from the co-design that suggest that lived experience and developmental stage are more important factors than age in relation to information needs, relevance, and acceptability.

The Gist web-based app does not include commenting features or social media integration other than sharing a quiz or an article directly with someone. This reduces the capacity for young people to interact negatively with each other and reflects our co-design research and previous studies suggesting that young people are unlikely to actively share any content relating to sexual health on social media [41]. Although the prototype design can be developed as a standalone, direct-to-consumer digital resource; we recommended that implementation be embedded within a multipronged health promotion approach such as service-delivered workshops. This is in response to the positive reaction that our co-design workshops were met with and the participants explicitly stating that they valued the opportunity to direct conversations with adults on sex and relationships. Scaffolding the digital product with service-delivered face-to-face workshops would also act as an entry point to the digital solution to enhance reach and engagement, making a digital solution more feasible.

## Discussion

### Principal Findings

The findings of our co-design workshops identify unmet sexual health and pornography literacy information needs among our users, their information-seeking patterns, and preferences for a digital resource. Through this process, we were able to develop The Gist prototype with young co-designers. The detailed and revealing nature of our findings suggests that using participatory research methods such as co-design can overcome sociocultural and structural barriers to engaging vulnerable young people in research on sensitive health issues. Some notable findings from this cross-disciplinary approach are discussed in the following sections in the context of current literature and directions for future research.

### Unmet Information Needs and the Limitations of Current Pornography Literacy Approaches

Most formal pornography literacy programs focus on the critical awareness of pornographic content and the industry [32]. We found that our users understood pornography as obviously unrealistic and could already critique its messages. Consistent with findings from the study by Dawson et al [33], a key message of this study is that effective pornography literacy programs must expand their focus on problematic messages from pornography and go beyond critical media literacy to include alternative information to help young audiences to navigate sexuality and relationships in their lives.

Few resources exist that directly contrast information about sex with messages from pornography and society [50-52]. There are some notable exceptions that include a cross-section of relevant topics [53]. However, the usability and acceptability of the resource among subgroups of young people are unknown. Our findings suggest that there is potential to embed pornography literacy within practical information about ethical sex and relationships to help young people transfer critical literacy into alternate choices and preferences in real life, but that this approach must be evaluated [49]. Any approach must balance sex-positive language and framing with acknowledgment of the negative lived experiences of some young people who require sensitively delivered information acknowledging these experiences. Although we included safeguarding approaches such as progressive disclosure and trauma-informed language, further research would strengthen the approach to pornography literacy programs.

A large body of the literature has identified the information deficit that young people experience as a result of current education approaches that fail to adequately teach about pleasure, sexual discovery, or self-representation [21,54,55]. We found that for vulnerable young people, there were critical unmet information needs in relation to the basics of navigating sex and relationships that compounded fetishized representations in pornography [33]. Thus, having relevant and accessible alternative information may be even more vital for vulnerable young people whose basic needs may not be met by schools, parents, or peers [25]. Importantly, our findings reinforce the need for longitudinal research to understand the specific impacts of pornography on the lives of subgroups of young people, including disengaged LGBTQI+ and CALD groups, if their needs are to be met.

### Co-Design and Digital Health Promotion

Research has illustrated limitations to traditional approaches to intervention design, which involve engagement with target users in research to inform content development by subject matter or technical experts rather than by engaging end users throughout the process as equal partners in design [39,56,57]. Where topics are particularly complex or sensitive, asking young people to engage in one-on-one interviews or even focus groups may be ethically sensitive and/or increase social desirability bias. Using creative and generative activities, we observed an extremely high level of engagement among young people, even on difficult topics that sometimes included traumatic experiences. Creating a safe space where participants could use creation to express an experience, rather than having to speak directly about it, enabled them to have their perspectives included and to better understand the underlying issues and design a more inclusive solution. The study provides an example of how co-design methods are responsive to sensitive and complex health issues and are particularly suitable for designing with rather than for young people [40,43]. More work needs to be done evaluating the effectiveness of co-designed interventions compared with evidence-based expert-designed approaches.

### Limitations

These findings should be considered within the context of study limitations. Co-design with *extreme* or *hard-to-reach* users generates products that can appeal to broader populations [39,43,58]; however, co-design research needs to be undertaken to test and adapt the prototype for specific audiences. We could not report quotes and attributions in this publication because of ethical concerns that service providers who helped with recruitment may be able to identify participants. Despite these limitations, the study provides important findings relating to pornography literacy and intervention design because of high-quality, in-depth, and reoccurring engagement with this vulnerable population.

### Conclusions

The study findings include important recommendations for the conceptualization of online pornography literacy programs and opportunities for cross-disciplinary research to address complex and emerging health issues. If pornography literacy programs focus on pornography without providing young people with alternative information about sex and relationships, they may not be effective in reducing harm. This study highlights the potential for pornography literacy to be embedded within real and practical information about having sex, navigating sexuality, and healthy relationships. The cross-disciplinary approach reported in this study demonstrates that it is possible to collaboratively co-design a pornography literacy resource combining the needs and wants of vulnerable young people with best practice in design and health promotion.

## Abbreviations

CALD: culturally and linguistically diverse
LGBTQI+: lesbian, gay, bisexual, transsexual, queer, and intersex plus
UX: user experience
